# Response of Fumaric Acid Addition on Methanogenesis, Rumen Fermentation, and Dry Matter Degradability in Diets Containing Wheat Straw and Sorghum or Berseem as Roughage Source

**DOI:** 10.5402/2012/496801

**Published:** 2012-04-17

**Authors:** S. K. Sirohi, Poonam Pandey, Navneet Goel

**Affiliations:** Nutrition Biotechnology Laboratory, Dairy Cattle Nutrition Division, National Dairy Research Institute, Haryana 132001, India

## Abstract

An *in vitro* incubation system was used to evaluate effect of supplementation of fumaric acid at 0, 5, 10, and 15 mM concentration in high-, medium-, and low-fiber wheat straw containing total mixed diets with sorghum (*Sorghum vulgare*) and berseem clover (*Trifolium alexandrinum L.*) on rumen fermentation, methane production, and gas kinetics parameters. Three types of diets were prepared with different roughage and concentrate ratio (80 : 20, 50 : 50, and 20 : 80) by taking the representative samples. The roughage part composed of wheat straw (70 parts) and sorghum (30 parts) or berseem (30 parts) and the concentrate part composed of maize (33%), GNC (21%), mustard cake (12%), wheat bran (20%), deoiled rice bran (11%), mineral mixture (2%), and salt (1%). Fumaric acid was added in incubation medium to achieve final concentration of 0, 5, 10, and 15 mM. All the treatment combinations were arranged in 4 × 3 factorial designs with three replicates. It was concluded from the study that fumaric acid addition (5–15 mM) in diets varying in roughage to concentrate ratio significantly (*P* ≤ 0.05) reduced the methane production without affecting dry matter digestibility and maximum reduction was noticed at 5 mM concentration.

## 1. Introduction

Many of compounds have been tested as potential feed additives for ruminants on the basis of their direct or indirect effects on CH_4_ production in the rumen. These compounds include ionophores, halogenated CH_4_ analogues, and unsaturated fatty acids [1, 2]. Other approaches are to reduce protozoal population which is believed to have symbiotic relationship with methanogens by using different compounds [3]. Another strategy of diverting H_2_ from CH_4_ production is to increase alternative electron sink metabolic pathways to dispose of the reducing power [3–5]. Fumarate and malate are key intermediates in the succinate-propionate pathway, in which malate is dehydrated to fumarate and fumarate reduced to succinate, which is then decarboxylated to propionate. Reducing equivalents are consumed in the reduction of fumarate to succinate, and hence both fumarate and malate have been shown to compete successfully for in the rumen with reduced methane production both *in vitro* and *in vivo* conditions. Therefore, present study was planned to evaluate the effect of fumaric acid on diets containing wheat straw and sorghum or berseem as roughage source at different concentration generally used in Indian conditions for various categories of dairy animals.

## 2. Materials and Methods

### 2.1. Feeds and Experimental Design

 To evaluate the response of fumaric acid, three diets were prepared by taking different roughage and concentrate ratio of 50 : 50, 20 : 80, and 80 : 20 on dry matter basis. The roughage part composed of wheat straw (70 parts) and sorghum (30 parts) or berseem (30 parts) and the concentrate part composed of maize (33%), groundnut cake (21%), mustard cake (12%), wheat bran (20%), deoiled rice bran (11%), mineral mixture (2%), and salt (1%). Fumaric acid (Sigma-Aldrich, EC203-743-0) was added in incubation medium to achieve final concentration of 0, 5, 10, and 15 mM prepared in distilled water (DW), and required quantities in equal volumes were added in both control (only DW) and treatments. All the treatment combinations were arranged in 4 × 3 factorial arrangements in randomized block design with three replicates. A set was incubated devoid of substrate with and without fumaric acid which served as blanks for particular treatment and values were corrected for different parameters with these blanks.

### 2.2. Preparation of Inoculum

 Rumen liquor was collected from a fistulated male buffalo maintained on a standard diet (60 parts roughage : 40 parts concentrate) before morning feeding into a pre-warmed insulated flask and brought into the laboratory. The rumen liquor was filtered through four layers of muslin cloth and then the required amount of filtered rumen liquor used as a source of inoculums. Required approval was obtained from Institutional Animal Ethics Committee prior to conduct experiments.

### 2.3. *In Vitro* Gas Production

 The substrate was milled to pass through 1 mm sieve, and 200 ± 10 mg was weighed in glass syringes of 100 mL capacity. 100 mL glass syringes were used for *in vitro* gas production technique [6]. The 30 mL incubation medium was dispensed anaerobically in each syringe. Syringes were incubated at  39 ± 0.50°C for 24 h. Before incubation, fumaric acid solution injected as per the dose by small syringe into 100 mL syringes individually. Syringes were pre-warmed (39 ± 1°C) prior to the addition of 30 mL buffered rumen liquor into each syringe under CO_2_ flushing. Plungers of syringes were applied with petroleum jelly for smooth movement and stop any leakage. Syringes were closed using clamps and were incubated for 24 h in case of *in vitro* dry matter digestibility (IVDMD), total volatile fatty acids (TVFAs), individual volatile fatty acids (IVFAs), and methane, while incubated for 96 h (in sequential incubation for 0, 1, 2, 3, 6, 9, 12, 24, 30, 36, 48, 60, 72, and 96 h) in case of gas kinetics study.

### 2.4. Total Gas Production and Methane Estimation

After 24 h incubation, total gas production was estimated by the displacement of piston during incubation. The gas produced due to fermentation of substrate was calculated by subtracting gas produced in blank syringe (containing no substrate, but only the inoculum and buffer) from total gas produced in the syringe containing substrate and inoculum and buffer. For methane estimation, representative gas was sampled from the headspace of syringe in an airtight syringe and injected into Nucon-5765 gas chromatograph equipped with flame ionization detector (FID) and stainless steel column packed with Porapak-Q. The gas flow rates for nitrogen, hydrogen, and air were 30, 30, and 300 mL/min, respectively. Temperature of injector oven, column oven, and detector were 40, 50, and 50°C, respectively. A 50/50 mixture of methane and carbon dioxide (Spancan; Spantech Products Ltd., England) was used as a standard.

### 2.5. Partitioning Factor (PF) and Microbial Biomass Yield (MBM)

 The PF is calculated as the ratio of substrate truly degraded *in vitro *(mg) to the volume of gas (mL) produced. The MBM yield was calculated by using the degradability of substrate and gas volume and stoichiometrical factor [7]:

(1) $$\begin{array}{cc} & Microbial\;mass\;\left(mg\right)\\ & \;=\;Substrate\;truly\;degraded\\ & \;\;-\;\left(gas\;volume\;\times \;stoichiometrical\;factor\right), \end{array}\;\;$$

where the stoichiometrical factor used was 2.25.

### 2.6. Gas Production Kinetics

 Kinetics of gas production was calculated using a nonlinear model [8]. The NLIN procedure of Sigma stat 3.11 was used to fit the following model: *p* = *b*  [1 − *e* − *c*(*t*)], where *p* is the gas production rate at time* t*,* b* is the potential gas production (mL), and *c* is gas production rate constant (mL/h) of *b* and *t* is the time of incubation (h). The total gas production kinetics was carried out in different treatment combinations incubated as per procedure mentioned above for different intervals that is, 0, 1, 2, 3, 6, 9, 12, 24, 36, 48, 60, 72, and 96 h. The potential gas production and rate of gas production was calculated by fitting the modified equation [8].

### 2.7. Rumen Fermentation Parameters

 The supernatant of each syringe including that of blank was used for NH_3_-N estimation. Supernatant (5 mL) was mixed with 1 N NaOH (2 mL) and steam passed on this using KEL PLUS-N analyzer (Pelican, India), and the NH_3_ evolved was collected in boric acid solution having mixed indicator and titrated against N/100 H_2_SO_4_. TVFA concentration (mmol/100 mL) in the supernatant was estimated [9] in the supernatant. At the end of incubation (24 h), 1 mL of the supernatant was treated with 25% meta-phosphoric (4 mL) and kept for 3-4 h at ambient temperature [10]. Thereafter, it was centrifuged at 3000 rpm for 10 minutes, and clear supernatant was collected and stored at −20°C until analyzed. IVFA was estimated using gas chromatograph (Nucon 5700, India) equipped with flame ionization detector (FID) and stainless steel column (length 4′; o.d (1/8)′′; i.d 2 mm) packed with chromosorb-101. Temperature of injection port, column and detector was set at 200, 180, and 210°C, respectively. The flow rate of carrier gas (nitrogen) through the column was 40 mL/min; and the flow rate of hydrogen and air through FID was 30 and 300 mL/min, respectively. Sample (2 *μ*L) was injected through the injection port using Hamilton syringe (10 *μ*L). Individual VFAs of the samples were identified on the basis of their retention time and their concentration (mmol) and calculated by comparing the retention time as well as the peak area of standards after deducting the corresponding blank values.

### 2.8. Protozoa Counting

 For protozoal count, one milliliter of the fermentation fluid was diluted with 1 mL of formalin (18.5% formaldehyde) and 3-4 drops of brilliant green and then incubated for 24 hours at room temperature. The stained protozoa were diluted (if needed) and counted by Haemocytometer [11].

### 2.9. *In Vitro* True DM Degradability, Proximate Principles, and Cell Wall Constituents

 To estimate *in vitro* true DM, degradability of feed sample in each syringe was estimated after end of incubation period [12]. The proximate principles [13] and cell wall constituents [14] were also estimated for substrate.

### 2.10. Statistical Analysis

 Experimental data of different parameters were analyzed in 4 × 3 with three replicates factorial arrangement in randomized block design with three replicates for analysis of variance [15]. The effects of different diet and doses of fumaric compared with control were tested using the factorial arrangement in complete randomized block design in OPSTAT statistical software developed by Chaudhry Chran Singh Haryana Agriculture University, Hissar, Haryana, India. When the overall *F*-test was significant, differences between means and the control were declared significant at *P* ≤ 0.05 using the Fisher's Least Significant Difference (Critical Difference).

## 3. Results

**Figure 1:**
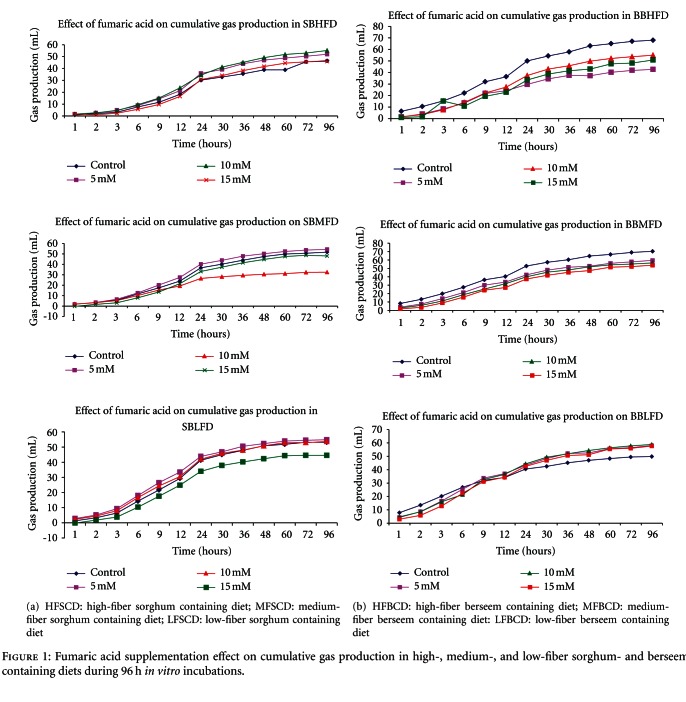
Fumaric acid supplementation effect on cumulative gas production in high-, medium-, and low-fiber sorghum- and berseem-containing diets during 96 h *in vitro* incubations.

 The chemical composition of different diets was presented in Table 1. As expected, the CP and NDF contents were increased with increasing the level of roughage in diets. Ether extract was also found highest in LFD (60C : 40R) and lowest in HFD (40C : 60R). The effects of fumaric acid addition on *in vitro* rumen fermentation pattern and methane production of different diets were shown in Table 2 to Table 5, respectively. In all treatment combinations, the pH was remained relatively stable at near range in sorghum-based diets and in berseem-based diets and statistically remained similar in all diets. Results of digestible dry matter (DDM) were increased by addition of fumaric acid mainly in diets containing berseem; however, DDM values almost remained similar in sorghum-based diets. The maximum DM digestibility values were noticed in low-fiber diets at 10 mM dose. DM digestibility values were found significantly (*P* ≤ 0.05) increased at 5 mM and 10 mM dose of fumaric acid in all type of diets; however, at 15 mM dose, a reduction of DM digestibility was found in all high-, medium-, and low-fiber diets, respectively (Table 2). Similarly, the PF and MBM values were increased (*P* ≤ 0.05) with supplementation of fumaric acid at different concentration in all dietary treatment combinations in berseem-based diets. The differences of PF and MBM values among different doses of fumaric acid and diets were found significant (*P* ≤ 0.05). The results related with methane production showed decreasing trend due to fumaric acid supplementation in high-, medium-, and low-fiber berseem-based diets, respectively. Methane (mL/g DM) reduced from 44.08 to 22.45 in HFD, 53.68 to 27.18 in MFD, and 58.61 to 30.66 in LFD, respectively. The similar trend was noticed in case of methane (mM/g DM) which was also significantly (*P* ≤ 0.05) reduced in different treatment combinations in all the three berseem-based diets (Table 2). In case of methane, the maximum decrease was noticed at 15 mM in HFD, and similar in case of MFD; however, in LFD, maximum reduction was found at 5 mM dose in comparison to other doses of fumaric acid. The values of methane (mL/g DM and mM/g DM) among treatment and diets found to be significantly different at *P* ≤ 0.05. The results with respect to VFA production indicated that TVFA concentration was increased due to addition of fumaric acid at different concentration in all diets and maximum value was noticed at 15 mM dose in LFD and the minimum value was noticed at 5 mM dose in HFD in all the diets. TVFA content increased significantly at all levels of fumaric acid supplementation in high-, medium-, and low-fiber berseem-based diets in comparison to control (Table 3). The value of acetate production remained similar in all the diets; however, propionate production was significantly increased (*P* ≤ 0.05) by supplementation of fumaric acid at different concentrations in all berseem-based diets. The propionic acid production ranges from 1.00 to 2.41 mM/100 mL in different dietary treatment combinations, and the maximum values were noticed at highest concentration of fumaric acid, that is, 15 mM in comparison to other concentration of fumaric acid and differences between berseem-based diets and treatments were statistically significant (*P* ≤ 0.05). The A : P ratio was also decreased with increase in concentration of fumaric acid in most of treatment combinations in berseem-based diets (Figure 1). Butyric acid was significantly decreased due to fumaric acid supplementation in all diets, and the lowest concentration was found in case of HFD and highest concentration in case of LFD. The differences of butyrate production among the diet in different treatment remained statistically significant at *P* ≤ 0.05. Ammonia nitrogen and protozoal number was also decreased due to supplementation of fumaric acid in different dietary treatment combinations, and differences among the treatments were statistically significant at 5% (Table 3).

 Results of sorghum-based diets are presented in Tables 4 and 5, and the results of *in vitro *study indicated that values of IVDDM were significantly reduced at 15 mM in HFD in comparison to other treatment combinations in medium and low-fiber sorghum-based diets. In HFD, DDM was significantly (*P* ≤ 0.05) decreased from 133.00 mg in control to 118.00 mg at 15 mM dose.

 The PF and MBM yield were increased significantly (*P* ≤ 0.05) with fumaric acid supplementation at different doses in all dietary treatment combinations (Table 4). In HFD, highest PF (4.59) and highest MBM yield (68.96 mg) was found at 5 mM dose, as compared to control PF (4.07) and MBM yield (59.50 mg). In MFD, PF and MBM yield was also found highest at 5 mM dose, the PF and MBM yield was 3.80 and 61.13 mg (in control) and 4.21 and 69.17 mg at 5 mM dose, respectively. But in case of LFD, highest PF and MBM yield was found at 15 mM dose and it was 4.15 and 71.58 mg, respectively. A significant methane reduction was seen in all types of diet and treatment combinations due to addition of fumaric acid in sorghum-based diets. In HFD, methane (mL/gm DM) reduced from 39.07 mL to 27.26 mL in HFD, 45.68 mL to 33.46 mL in MFD, and 49.72 mL to 35.66 mL in LFD. Similar trend in methane reduction was also noticed in methane (mM/g DM). A significant effect of fumaric acid supplementation in sorghum-based diets was found on TVFA (mM/100 mL) concentration. In HFD, MFD, and LFD, the highest concentration of TVFA was found at 15 mM dose, that is, 6.62, 7.15, and 7.27 mM, respectively. Acetate concentration (mM/100 mL) did not differ significantly among treatment diets, whereas propionate (mM/100 mL) concentration was increased significantly (*P* < 0.05) due to fumaric acid supplementation (Table 3). The butyric acid (mM/100 mL) concentration was significantly decreased due to fumaric acid treatment in all three diets; lowest concentration was found in HFD, and highest was found in LFD. A significant decrease in NH_3_-N concentration (mg/100 mL) was observed due to fumaric acid treatment. In HFD, the highest decrease in NH_3_-N concentration was at 10 mM dose, that is, 19.13 to 16.80 mg/100 mL, in MFD, highest decrease was at 5 and 15 mM dose that is, 24.27 to 20.07 mg/100 mL, and, in LFD, it was at 10 mM dose, that is, 27.53 to 22.40 mg/100 mL. Protozoal number also decreased significantly (*P* ≤ 0.05) due to fumaric acid supplementation in different dietary treatment combinations in sorghum based diets.

Results related to gas kinetics in sorghum- and berseem-based diets presented in Table 6. Potential gas production (*b*) was increased due to addition of fumaric acid in sorghum-based diets, and increase was noticed up to 10 mM of fumaric acid supplementation. Similarly, the gas production rate constant (*c*) also increased in treatment combinations in comparison to control in high-, medium-, and low-fiber sorghum-based diets. The* b* values range from 32.18 to 57.11 mL in sorghum-based diets, but in case of berseem-based diets, the *b* values decreased with supplementation of fumaric acid at different concentration in comparison to control except in low-fiber diet. The gas production rate (*c*) decreased after fumaric acid treatment. In berseem-based diets,* b* value ranges from 40.85 to 66.46 mL and *c* values from 0.05 to 0.138 mL/h, respectively.

## 4. Discussion

 Results of the study revealed that effect of fumaric acid was dependent on nature of diets as seen with the digestible dry matter which increased in most of the treatment combinations with berseem-containing diets but effect was negligible with sorghum-based diets but little reduction in digestibility was noticed at high concentration (15 mM) of fumaric addition. The present findings were more or less in accordance with the studies in which increased digestibility of DM with fumaric acid and other organic acids in different diets were reported [5]. The main reason for improvement in digestibility might be due to increase in number of cellulolytic organisms which were benefited from the presence of methanogenic or other H_2_ utilizing bacteria as a result of interspecies H_2_ transfer [16]. Further, if hydrogen removal or uptake was increased by the addition of propionate precursors or dicarboxylic acids which might stimulate the growth of fibrolytic bacteria and hence enhance cellulose digestion [1, 3]. The addition of fumarate at different concentration in both diets (berseem and sorghum) resulted in significantly increased propionate production with different ratio of roughage and concentrate. Maximum concentration of propionate was observed at higher dosage of fumaric acid than lower dosages. Similar results were reported in different studies with organic acid supplementation [3, 5, 17, 18].These studies clearly indicated that fumaric acid provides an electron sink for metabolic hydrogen and helps in more propionic acid production. The addition of fumarate not only decreases CH_4_ production but also increased propionate, succinate, or both and slightly increased acetate and butyrate [2, 3].

 In present study, methane reduction was found maximum in all three types of berseem-containing diets than the sorghum diets. The conversion of glucose to acetate, propionate, and butyrate in the rumen results in an overall net release of reducing power. Much of this is used by methanogenic archaea to reduce CO_2_ to CH_4_, but H_2_ can also be used as a substrate in fumarate reduction [19]. As result H is used to reduce fumarate, there is a decrease in the availability of H for methanogenesis in the rumen which could decrease methane production. Fumarate, in the present study, found significantly (*P* ≤ 0.05) decreased methane production with all substrates in different ratio of roughage to concentrate, but the ability of methane reduction varies depending upon concentration of fumarate and type of diet [3, 20]. Ammonia concentration in present study was decreased due to addition of fumarate in most of the treatment combinations [20]. The PF of the diets is known as an index of microbial biomass synthesis efficiency [7], and the diet formulation to achieve higher PF would mean aiming for higher MBME *in vivo *[21, 22]. In present experiment, berseem-based diets had significant improvement effect on MBM yield which might be easily correlated with decrease ammonia concentration after addition of fumaric acid which means higher uptake of ammonia by mixed ruminal bacteria for microbial protein synthesis. Moreover, results of the gas kinetics showed that potential gas production was increased in sorghum-based diets but decreased in berseem-based diets correspondingly. The rate of gas production increased in sorghum-based diets and decreased in berseem-based diet which also related with digestibility of different diets formulated based on berseem and sorghum.

## 5. Conclusions

Overall, it was concluded from the results of the present study that fumaric acid supplementation in sorghum and berseem-based diets in different ratio of roughage to concentrate should able to modulate the rumen fermentation pattern and significantly decreased the methane production by diverting the hydrogen towards propionate production without affecting the digestibility adversely but digestibility of dry matter more or less affected by nature of proximate principles.

## Figures and Tables

**Table 1:** Chemical composition of berseem and sorghum containing diets.

Particulars (g/kg on DM basis)							
Diets	OM	CP	EE	NDF	ADF	HC	TA
Berseem-containing diets							
HFD (80R : 20C)	842.6	110.8	27.0	541.4	357.3	184.0	157.4
MFD (50R : 50C)	859.0	168.0	32.6	502.2	264.9	237.3	141.0
LFD (20R : 80C)	876.9	229.8	37.9	386.2	199.9	186.3	123.1

Sorghum-containing diets							
HFD (80R : 20C)	893.4	115.5	18.5	575.2	391.4	183.8	106.6
MFD (50R : 50C)	900.0	178.1	22.6	422.6	290.1	132.5	100.0
LFD (20R : 80C)	901.9	196.3	35.2	279.0	192.2	86.8	98.1

OM: organic matter; CP: crude protein; EE: ether extract; NDF: neutral detergent fiber; ADF: Acid detergent fiber; HC: hemicellulose; TA: total ash.

HFD: high-fiber diet; MFD: medium-fiber diet; LFD: low-fiber diet; R: roughage; C: concentrate.

*Roughage part composed of wheat straw (70 parts) and sorghum (30 parts) or berseem (30 parts).

**Table 2:** Supplementation effect of fumaric acid on digestibility in sorghum containing diets.

Parameters									
Sorghum-containing diets	Dose (mM)	pH	DDM (mg)	PF (mg TDMD/mL gas)	MBM (mg)	CH_4_ (mL/gm DM incubated)	CH_4_ (mM/g DM)	NH_3_-N (mg/100 mL)	Protozoa (×10^4 ^mL^−1^)
HFD (80R : 20C)	0	7.13	133.00	4.07	59.50	39.07	3.88	19.13	2.50
	5	7.19	135.33	4.59	68.96	33.32	3.31	21.47	1.50
	10	7.13	133.33	4.00	58.33	30.62	3.05	16.80	1.25
	15	7.15	118.00	4.00	51.63	27.26	2.71	20.07	1.25

MFD (50R : 50C)	0	7.14	150.00	3.80	61.13	45.68	4.55	24.27	1.63
	5	7.19	148.67	4.21	69.17	38.72	3.85	20.07	1.75
	10	7.24	148.33	4.03	65.46	34.86	3.47	26.13	1.75
	15	7.15	139.33	3.89	58.71	33.46	3.33	20.07	0.75

LFD (20R : 80C)	0	7.21	157.67	3.73	62.42	49.72	4.94	27.53	0.75
	5	7.16	158.00	3.97	68.38	42.77	4.25	24.27	0.50
	10	7.15	159.00	3.91	67.50	37.83	3.76	22.40	1.00
	15	7.14	156.33	4.15	71.58	35.66	3.55	28.00	1.50

SEM	Diets (D)	NS	1.55	0.04	1.29	0.33	0.03	1.09	NS
	Treatments (T)	NS	1.79	0.05	1.49	0.39	0.04	NS	NS
	D × T	NS	NS	0.09	2.59	NS	NS	NS	NS

HFD: high-fiber diet; MFD: medium-fiber diet; LFD: low-fiber diet; R: roughage; C: concentrate; DDM: digestible dry matter (mg); PF: partition factor (mg TDMD/mL gas); MBM: microbial biomass (mg); SEM: standard error of means.

**Table 3:** Supplementation effect of fumaric acid on rumen fermentation in Sorghum containing diets.

Sorghum containing diets	Dose	TVFA (mM)	Acetate (mM)	Propionate (mM)	Butyrate (mM)
HFD (80R : 20C)	0	51.5	34.2	11.1	6.3
	5	62.7	39.8	16.2	6.7
	10	63.0	38.7	18.1	6.3
	15	66.2	40.0	19.9	6.3

MFD (50R : 50C)	0	61.8	40.4	14.5	7.0
	5	58.8	38.5	15.3	5.0
	10	68.2	43.4	19.4	5.4
	15	71.5	42.2	22.1	7.2

LFD (20R : 80C)	0	64.5	43.8	12.7	7.9
	5	70.3	43.2	19.5	7.6
	10	71.0	42.4	21.5	7.1
	15	72.7	41.9	23.0	7.7

SEM	Diet (D)	1.04	0.69	0.45	0.19
	Treatment (T)	1.20	NS	0.52	0.22
	D × T	NS	1.38	NS	0.04

HFD: high-fiber diet; MFD: medium-fiber diet; LFD: low-fiber diet; R: roughage; C: concentrate; TVFA, total volatile fatty acids (mM); H: hydrogen; SEM: standard error of means.

**Table 4:** Supplementation effect of fumaric acid on digestibility in berseem-containing diets.

Parameters									
Berseem-containing diets	Dose (mM)	pH	DDM (mg)	PF (mg TDMD/mL gas)	MBM (mg)	CH_4 _(mL/gm DM incubated)	CH_4_ (mM/g DM)	NH_3_-N (mg/100 mL)	Protozoa (×10^4 ^mL^−1^)
HFD (80R : 20C)	0	7.20	100.33	2.85	21.21	44.08	4.38	22.87	2.58
	5	7.20	130.33	4.58	65.84	26.23	2.61	14.47	2.83
	10	7.19	134.33	4.94	73.21	24.19	2.40	15.40	0.92
	15	7.14	118.00	5.39	67.38	22.45	2.23	15.87	1.25

MFD (50R : 50C)	0	7.17	105.33	2.47	9.33	53.68	5.34	21.00	2.58
	5	7.15	145.33	4.74	76.34	30.16	3.00	19.60	1.42
	10	7.23	140.00	4.44	69.13	29.15	2.90	17.27	1.50
	15	7.16	126.67	4.84	66.67	27.18	2.70	20.07	1.92

LFD (20R : 80C)	0	7.26	137.33	2.99	33.84	58.61	5.83	27.07	2.83
	5	7.19	139.67	4.64	70.67	30.66	3.05	23.80	0.83
	10	7.18	158.00	4.48	78.50	31.07	3.09	22.40	1.42
	15	7.18	120.67	3.54	44.42	35.72	3.55	22.87	1.25

SEM	Diet (D)	NS	4.46	NS	NS	1.45	0.14	0.32	NS
	Treatment (T)	NS	5.15	0.19	4.96	1.67	0.17	0.37	0.27
	D × T	NS	NS	NS	NS	NS	NS	0.64	NS

HFD: high-fiber diet; MFD: medium-fiber diet; LFD: low-fiber diet; R: roughage; C: concentrate; DDM: digestible dry matter (mg); PF: partition factor (mg TDMD/mL gas); MBM: microbial biomass (mg); SEM: standard error of means.

**Table 5:** Supplementation effect of fumaric acid on rumen fermentation in berseem-containing diets.

Berseem-containing diets	Dose	TVFA (mM)	Acetate (mM)	Propionate (mM)	Butyrate (mM)
HFD (80R : 20C)	0	51.5	36.1	10.0	5.4
	5	53.2	34.8	13.7	4.7
	10	58.8	36.8	17.3	4.8
	15	61.3	37.5	19.1	4.7

MFD (50R : 50C)	0	57.0	40.0	10.6	6.5
	5	55.7	35.5	15.2	4.9
	10	60.5	38.1	16.7	5.7
	15	59.5	35.3	19.1	5.2

LFD (20R : 80C)	0	58.2	39.6	11.0	7.6
	5	54.0	35.4	13.4	5.2
	10	65.5	39.2	20.5	5.9
	15	73.3	42.7	24.1	6.5

SEM	Diet (D)	1.42	NS	0.49	0.15
	Treatment (T)	1.64	NS	0.56	0.18
	D × T	NS	NS	0.98	NS

HFD: high-fiber diet; MFD: medium-fiber diet; LFD: low-fiber diet; R: roughage; C: concentrate; TVFA: total volatile fatty acids (mM); H: hydrogen; SEM: standard error of means.

**Table 6:** Effect of fumaric acid on gas kinetics (96 h) in sorghum- and berseem-containing diets.

Regression model Orskov and Macdonald without lag									Equation: *F* = *b* × (1 − exp⁡ (−*c* × *x*))			
Diet type	HFD ( 80R : 20C)				MFD ( 50R : 50C)				LFD (20R : 80C)			

Dose (mM)	0	5 mM	10 mM	15 mM	0	5 mM	10 mM	15 mM	0	5 mM	10 mM	15 mM

Sorghum diet												
*b*	47.423	53.815	57.114	49.999	52.762	55.059	32.180	51.193	53.990	54.610	53.636	45.779
*c*	0.037	0.042	0.039	0.0351	0.048	0.052	0.069	0.041	0.058	0.070	0.064	0.055
*R* ^2^	0.990	0.993	0.994	0.984	0.998	0.996	0.997	0.989	0.995	0.997	0.998	0.989

Berseem diet												
*b*	65.945	40.854	54.731	49.620	66.459	56.360	54.919	52.590	46.454	54.463	56.077	54.699
*c*	0.066	0.068	0.052	0.050	0.082	0.073	0.064	0.057	0.138	0.095	0.084	0.079
*R* ^2^	0.992	0.986	0.997	0.975	0.981	0.987	0.994	0.994	0.961	0.981	0.987	0.986

*b*: potential gas production (mL); *c*: gas production rate constant (mL/h); *R* ^2^: regression coefficient; HFD: high-fiber diet; MFD: medium-fiber diet; LFD: low-fiber diet; R: roughage; C: concentrate.

## References

[1] Asanuma N, Hino T (2000). Activity and properties of fumarate reductase in ruminal bacteria. *Journal of General and Applied Microbiology*.

[2] Asanuma N, Iwamoto M, Hino T (1999). Effect of the addition of fumarate on methane production by ruminal microorganisms in vitro. *Journal of Dairy Science*.

[3] López S, Valdés C, Newbold CJ, Wallace RJ (1999). Influence of sodium fumarate addition on rumen fermentation *in vitro*. *British Journal of Nutrition*.

[4] Ungerfeld EM, Rust SR, Burnett R (2003). Use of some novel alternative electron sinks to inhibit ruminal methanogenesis. *Reproduction Nutrition Development*.

[5] Newbold CJ, López S, Nelson N, Ouda JO, Wallace RJ, Moss AR (2005). Propionate precursors and other metabolic intermediates as possible alternative electron acceptors to methanogenesis in ruminal fermentation *in vitro*. *British Journal of Nutrition*.

[6] Menke K, Steingass H (1988). Estimation of the energetic feed value obtained from chemical analysis and *in vitro* gas production using rumen fluid. *Animal Research and Development*.

[7] Blümmel M, Makkar HPS, Becker K (1997). *In vitro* gas production: a technique revisited. *Journal of Animal Physiology and Animal Nutrition*.

[8] Ørskov ER, McDonald I (1979). The estimation of protein degradability in the rumen from incubation measurements weighted according to rate of passage. *The Journal of Agricultural Science*.

[9] John A, Barnett G, Reid RL (1957). Studies on the production of volatile fatty acids from grass by rumen liquor in an artificial rumen—I. The volatile acid production from fresh grass. *The Journal of Agricultural Science*.

[10] Erwin ES, Marco GJ, Emery EM (1961). Volatile fatty acid analysis of blood and rumen fluid by gas chromatograph.. *Journal of Dairy Science*.

[11] Dehority BA (1984). Evaluation of subsampling and fixation procedures used for counting rumen protozoa. *Applied and Environmental Microbiology*.

[12] Van Soest PJ, Wine RH, Moore LA, Hill AGG Estimation of the true digestibility of forages by the *in vitro* digestion of cell walls.

[13] Association of Official Analytical Chemists (1995). *Official Methods of Analysis*.

[14] Van Soest PJ, Robertson JB, Lewis BA (1991). Methods for dietary fiber, neutral detergent fiber, and nonstarch polysaccharides in relation to animal nutrition. *Journal of Dairy Science*.

[15] Snedecor GW, Cochran WG (1968). *Statistical Methods*.

[16] Wolin MJ, Miller TL, Hobson PN, Stewart CS (1997). Microbe-microbe interactions. *The Rumen Microbial Ecosystem*.

[17] Callaway TR, Martin SA (1996). Effects of organic acid and monensin treatment on *in vitro* mixed ruminal microorganism fermentation of cracked corn. *Journal of Animal Science*.

[18] Carro MD, Ranilla MJ (2003). Effect of the addition of malate on *in vitro* rumen fermentation of cereal grains. *British Journal of Nutrition*.

[19] Russell JB, Wallace RJ, Hobson PN, Stewart CS (1997). Energy-yielding and energy consuming reactions. *The Rumen Microbial Ecosystem*.

[20] Bayaru Syuhei K, Toshihiko K (2001). Effect of fumaric acid on methane production, rumen fermentation and digestibility of cattle fed roughage alone. *Animal Science Journal*.

[21] Blümmel M, Lebzien P (2001). Predicting ruminal microbial efficiencies of dairy rations by *in vitro* techniques. *Livestock Production Science*.

[22] Blümmel M, Karsli A, Russell JR (2003). Influence of diet on growth yields of rumen miro-organisms *in vitro* and *in vivo*: influence on growth yield of variable carbon fluxes to fermentation products. *British Journal of Nutrition*.

